# Clinical and anatomical characteristics associated with obstructive sleep apnea severity in children

**DOI:** 10.1016/j.clinsp.2022.100131

**Published:** 2022-11-02

**Authors:** Maria Fernanda Bozzini, Renata C. Di Francesco, Letícia A. Soster

**Affiliations:** Faculdade de Medicina da Universidade de São Paulo (FMUSP), São Paulo, SP, Brazil

**Keywords:** Sleep, Sleep Apnea Syndromes, Polysomnography, Child, AASM, American Academy of Sleep Medicine, AF, Anterior Facial height, AHI, Apnea-Hypopnea Index, AT, Adenotonsillectomy, BMI, Bone Mass Index, FA, Facial Axis, FD, Facial Depth, Kv, Kilowatts, LFH, Lower Facial Height, Ma, Miliamps, MA, Mandibular Arch, MP, Mandibular Plane angle, OSA, Obstructive Sleep Apnea, PF, Posterior Facial height, PSG, Polysomnography, VERT, Vertical growth coefficient, WHO, World Health Organization

## Abstract

**Purpose:**

To determine the clinical and anatomical characteristics associated with obstructive sleep apnea severity in children with adenotonsillar hypertrophy.

**Methods:**

The authors conducted a cross-sectional multidisciplinary survey and selected 58 Brazilian children (4‒9 years old) with adenotonsillar hypertrophy, parental complaints of snoring, mouth-breathing, and witnessed apnea episodes. The authors excluded children with known genetic, craniofacial, neurological, or psychiatric conditions. Children with a parafunctional habit or early dental loss and those receiving orthodontic treatment were not selected. All children underwent polysomnography, and three were excluded because they showed an apnea-hypopnea index lower than one or minimal oxygen saturation higher than 92%. The sample consisted of 55 children classified into mild (33 children) and moderate/severe (22 children) obstructive sleep apnea groups. Detailed clinical and anatomical evaluations were performed, and anthropometric, otorhinolaryngological, and orthodontic variables were analyzed. Sleep disorder symptoms were assessed using the Sleep Disturbance Scale for Children questionnaire. All children also underwent teleradiography exams and Rickett's and Jarabak's cephalometric analyses.

**Results:**

The mild and moderate/severe obstructive sleep apnea groups showed no significant differences in clinical criteria. Facial depth angle, based on Ricketts cephalometric analysis, was significantly different between the groups (*p* = 0.010), but this measurement by itself does not express the child's growth pattern, as it is established by the arithmetic mean of the differences between the obtained angles and the normal values of five cephalometric measurements.

**Conclusions:**

The clinical criteria and craniofacial characteristics evaluated did not influence the disease severity.

## Highlights

• Untreated obstructive sleep apnoea in children may result in severe health consequences, including cardiovascular, metabolic, endocrine and growth abnormalities.
• Other sleep impairments may be associated with obstructive sleep apnoea, affecting children's quality of life.
• Polysomnography is the gold standard for diagnosis, but it is costly and not widely available.
• Other studies that contribute to the diagnosis process are relevant and of great importance.

## Introduction

Pediatric Obstructive Sleep Apnea (OSA) affects 1-5% of all children^1^ and several comorbidities associated with may directly affect a child's quality of life. The most common known comorbidities cited in the literature are enuresis, hyperactivity, inattention, aggressiveness, anxiety, excessive daytime sleepiness, cognitive deficits, and poor school performance.^2^, ^3^, ^4^

Adenotonsillar hypertrophy is the main risk factor for pediatric OSA^3^ and evidence supports Adenotonsillectomy (AT) as the first-line treatment for this condition.^5^^,^^6^ Accurate diagnosis is best accomplished by the integration of clinical history and data from physical inspection and examinations confirming the presence of the obstruction.^7^^,^^8^ Polysomnography (PSG) remains the gold standard examination, and the diagnosis is confirmed if the child presents one or more OSA signs or symptoms associated with more than one Apnea and Hypopnea event per hour (AHI) during sleep.^7^

PSG values before surgery are very important in identifying children with moderate or severe OSA because they are at a higher risk of developing surgical complications or residual apnea after AT.^3^ OSA in children is classified as mild when the AHI is between 1 and 5 and the minimal saturation index is between 85% and 92%, while moderate to severe OSA is defined by an AHI higher than 5 and minimal saturation index less than 85%, depending on the PSG global analysis and clinical evaluation.^9^^,^^10^ The American Academy of Pediatrics suggests that all children with OSA should undergo PSG before surgery,^11^ but the procedure is expensive, time-consuming, and not readily accessible.^7^^,^^3^

In addition to adenotonsillar hypertrophy, obesity and craniofacial morphology are also cited as important predisposing factors for pediatric OSA.^4^ Therefore, clinical and anatomical parameters play an important role in the diagnostic process. The aim of this study was to determine the clinical and anatomical characteristics associated with obstructive sleep apnea severity in children on a waiting list for AT and help clinicians manage the surgical risks and post-surgical follow-up.

## Materials and methods

This cross-sectional interdisciplinary survey was conducted at the Otorhinolaryngology and Pediatrics Department of the University of São Paulo Medical School, São Paulo, Brazil. Children were recruited, and their parents agreed to participate by signing the informed consent form approved by the Institution's Committee on Ethics and Research.

### Study sample

Children were consecutively selected from the waiting lists for AT between August 2015 and January 2019. The sample was composed of 58 Brazilian children (4.0‒9.0 years old) with nasopharynx obstruction (> 60%), tonsil enlargement (Brodsky's grades^12^ 2, 3 or 4), parental complaints of snoring (> 3 nights per week), mouth-breathing, and/or witnessed apnea episodes. All children were surgical candidates for AT.

The authors targeted a population that was older than 4.0 years of age to ensure cooperation during the examinations and younger than 9.0 years of age to avoid the effects of pubertal growth spurts on the study outcomes.

Standardized PSG was performed using the 2014 American Academy of Sleep Medicine (AASM) criteria: AHI ≥ 1 and/or minimal saturation < 92%.^4^ Three children were excluded because they showed AHI < 1 and/or minimal saturation > 92% in PSG. The sample was then classified^9^ into the mild OSA group (33 children) and the moderate/severe OSA group (22 children), all candidates for AT. The authors were not able to recruit a control group because of ethical reasons for performing the exams on children without symptoms.

### Inclusion criteria

• Adenoid enlargement and tonsil enlargement
• Surgical candidate for AT
• Diagnosed with OSA defined as:

Parental report of snoring (an average of > 3 nights per week), mouth-breathing, and/or witnessed apnea episodes.

Nocturnal PSG confirming AHI ≥ 1 and/or minimal saturation < 92%.

### Exclusion criteria

• Known genetic, craniofacial, neurological, or psychiatric conditions likely to affect the airway, cognition, or behavior.
• Parafunctional habit (digital sucking or pacifier), early dental loss, and/or receiving orthodontic treatment.

### Clinical and anatomical analysis

A detailed clinical and anatomical analysis was performed. Age, gender, weight, and height were used to determine the BMI z-score using the World Health Organization (WHO) software. On the basis of their BMI z-scores, children were categorized as thin, eutrophic, overweight risk, overweight, or obese. All children underwent a systematic otorhinolaryngological examination. The adenoid size was determined by evaluating the percentage of nasopharyngeal obstruction observed in 3.2-mm ENT-P nasal fiberoptic endoscopy (Machida®, Japan). Tonsil size was determined by using Brodsky's criteria.^12^ Tonsil and adenoid evaluation were realized by two experienced otorhinolaryngologists.

Overnight PSG was performed using the Embla N7000 (Natus Neurology®, EUA) without sedation or sleep deprivation. Electrophysiological and cardiorespiratory parameters were recorded on a computerized system RemLogic (version 3.2, Natus Neurology®, USA). Sleep stages, hypopnea, central apnea, obstructive apnea, or mixed events were scored according to the 2014 AASM criteria.^4^

To assess the symptoms of sleep disorders, parents filled out the SDSC questionnaire,^13^ which has been already validated for the Portuguese language.^14^ The 26 questions are grouped into six subscales representing the most common sleep disturbances: disorders in initiating and maintaining sleep, disorders of breathing during sleep, disorders of arousal, disorders of sleep-wake transition, disorders of excessive somnolence, and nocturnal hyperhidrosis.

The orthodontic evaluation consisted of dental and facial clinical analysis and was realized by an experienced orthodontist. Children were instructed to maintain teeth in occlusion with lips at rest and the following variables were analyzed: absence of lip sealing (yes/no), facial profile (plane/convex), increased overjet (> 3 mm),^15^ distocclusion (Angle Class II^16^ or Baume distal step),^17^ and transversal arch relationship (normal/ unilateral crossbite or bilateral crossbite). The evaluation of facial profile is a routine part of the examination of an orthodontic patient and for that is necessary a cephalometric analysis which provides an exact depiction of craniofacial morphology. All children underwent teleradiography for cephalometric analysis. Radiographs were taken using Orthophos XG 5 DS Ceph (Sirona®, Brazil) regulated to 12 mA, 90 kV, and 0.30 s of time exposure. All children were oriented in the natural head position.

**Fig. 1:**
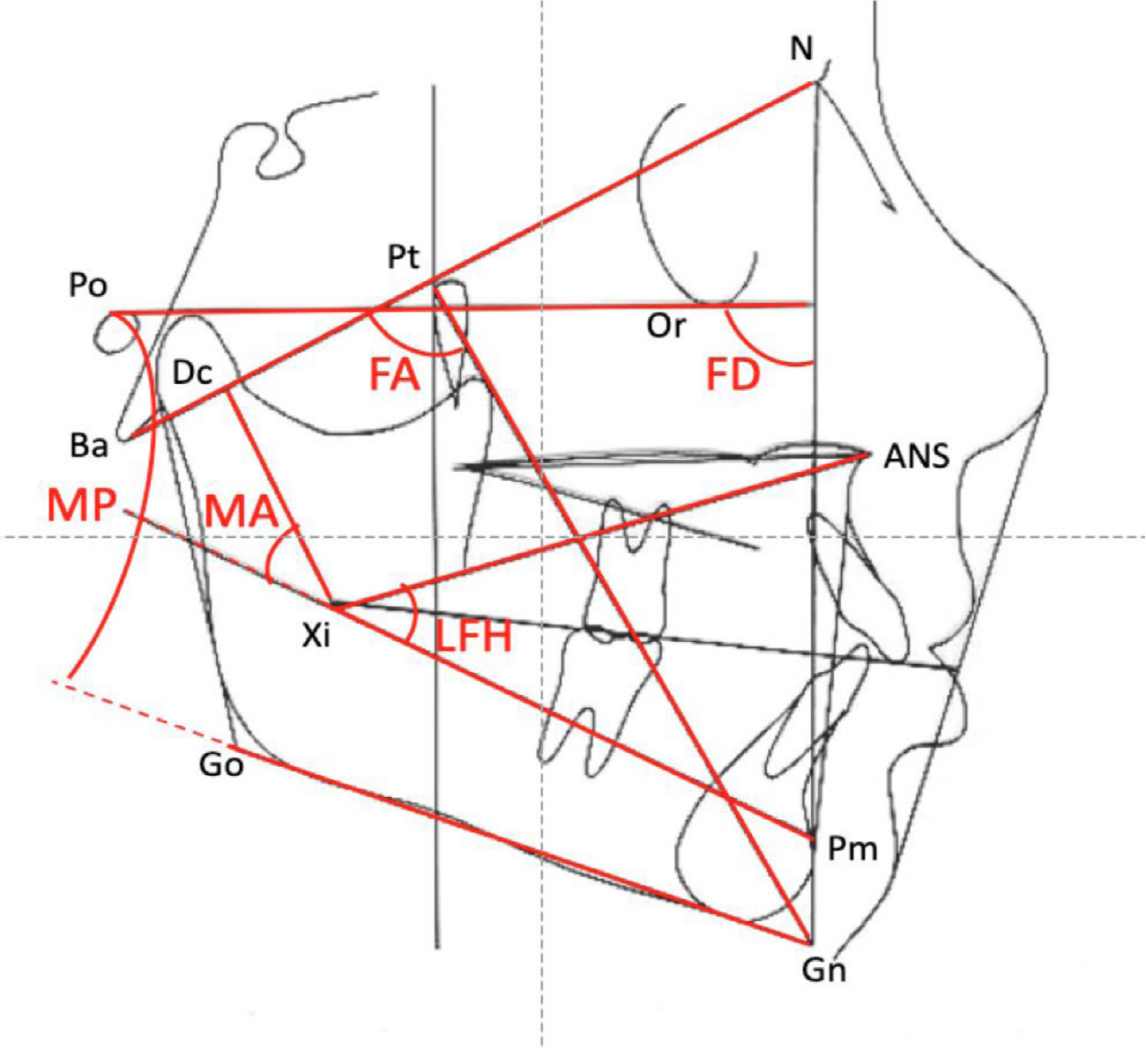
Rickett's cephalometric analysis- facial axis (FA), facial depth (FD), mandibular plane angle (MP), lower facial height (LFH) and mandibular arch (MA).

Radiographs were digitized and cephalometric analysis was performed using Easy Ceph (Anne Solutions®, Brazil) software. Rickett's cephalometric analysis^18^ included the following skeletal craniofacial measurements: Facial Axis (FA), Facial Depth (FD), Mandibular Plane angle (MP), Lower Facial Height (LFH), Mandibular Arch (MA), and Vertical Growth Coefficient (VERT). The facial pattern was established using the VERT index, which is calculated by determining the arithmetic mean of differences between the obtained and normal values of the measurements cited above, represented in Fig. 1. On the basis of the growth tendency, the children were then classified into mesofacial, dolichofacial (vertical), or brachyfacial (horizontal) groups.

**Fig. 2:**
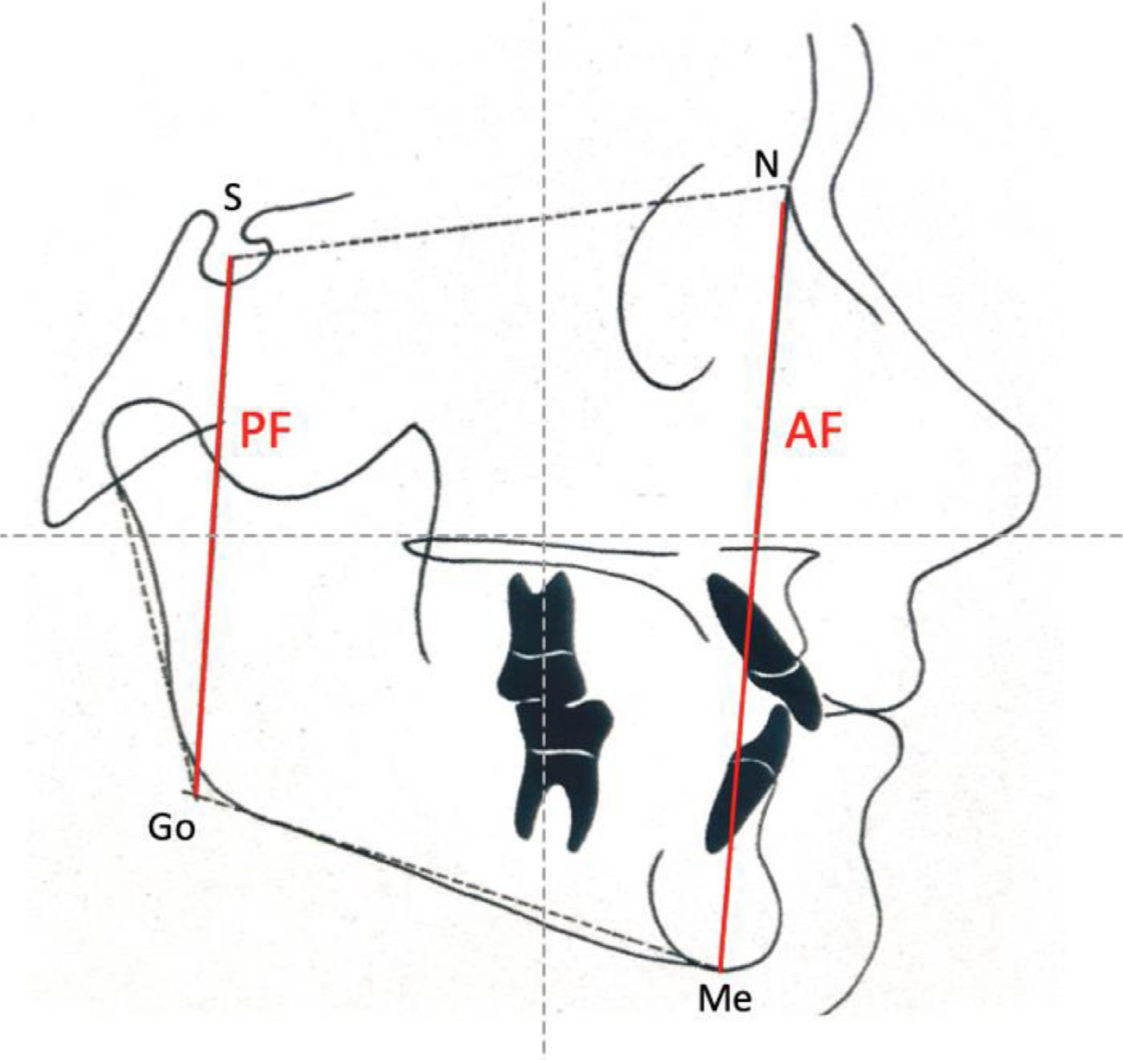
Jarabak's cephalometric analysis- posterior facial height (PF) and anterior facial height (AF).

Jarabak's cephalometric analysis^19^ included the following skeletal craniofacial measurements: Posterior Facial height (PF), Anterior Facial height (AF), and the Jarabak's coefficient percentage (PF/AF ×100), represented in Fig. 2. Children were classified into neutral, hypodivergent (horizontal), or hyperdivergent (vertical) growth tendency patterns according to the percentage.

### Statistical analysis

Categorical variables were described using absolute and relative frequencies and continuous variables were described using summary statistics (mean, standard deviation, median, minimum, and maximum).

Comparisons between categorical variables in the mild OSA and moderate/severe OSA groups were verified using Chi-Squared or exact tests (Fisher's exact test or likelihood-ratio test). Comparisons between continuous variables in the mild OSA and moderate/severe OSA groups were performed using Student's *t*-test. Odds ratios of each variable of interest were estimated with the respective 95% Confidence Intervals by using simple logistic regression. A multiple logistic regression model was estimated by selecting variables that showed *p* < 0.10 in the bivariate tests. Tests were performed with a 5% significance level.

## Results

The PSG factors and OSA stratification for all children are described in Table 1. The mean AHI value was 4.8 ± 7.6, with an average minimal oxyhemoglobin saturation (min Sat O_2_) of 86.8% ± 5.2%. Thus, 33 (60%) children were classified into the mild OSA group and 22 (40%) were classified into the moderate/severe OSA group.

**Table 1:** Polysomnography variables and obstructive sleep apnea classification for all children.

Variables	Description (*n* = 55) %
AHI	
Mean ± SD	4.8 ± 7.6
Median (min‒max)	2.3 (0.2‒48.7)
Minimal Sat O_2_ (%)	
Mean ± SD	86.8± 5.2
Median (min‒max)	88 (72‒95)
Mean Sat O_2_ (%)	
Mean ± SD	95.2± 1.8
Median (min‒max)	95 (90.6‒98)
Snoring during the exam	
No	30 (54.5)
Yes	25 (45.5)
OSA classification	
Mild	33 (60)
Moderate	13 (23.6)
Severe	9 (16.4)

Continuous variables were mean, standard deviation, median, minimum and maximum, and categorical variables were absolute and relative frequencies.

AHI, Apnea-Hypopnea Index; Sat O_2_, Blood Oxyhemoglobin Saturation; OSA, Obstructive Sleep Apnea.

Table 2 presents the demographic, anthropometric, and otorhinolaryngological data for both groups. The variables age, sex, BMI z-score, and adenoid and tonsil size were not significantly different between the groups. Sleep disorder symptoms associated with pediatric OSA were assessed using the SDSC questionnaire,^13^ and none of the six subscales showed significant differences between groups, as shown in Table 3.

**Table 2:** Demographic, anthropometric, and otorhinolaryngological findings for the mild OSA and moderate/severe OSA groups.

Variables	OSA		OR	95% CI		p-value
	Mild	Moderate/ Severe		Inferior	Superior	
Age (years)			1.37	0.96	1.97	0.084^a^
Mean ± SD	5.5± 1.4	6.2± 1.7				
Median (min‒max)	5 (4‒9)	6 (4‒9)				
Sex						0.375
Male	17 (54.8)	14 (45.2)	1.00			
Female	16 (66.7)	8 (33.3)	0.61	0.20	1.83	
Z score classification, n (%)						0.180^b^
Thin/Eutrophic	22 (62.9)	13 (37.1)	1.00			
Overweight risk/Overweight	9 (69.2)	4 (30.8)	0.75	0.19	2.94	
Obese	2 (28.6)	5 (71.4)	4.23	0.72	25.02	
Adenoid enlargement (%)			1.00	0.96	1.03	0.866^a^
Mean ± SD	69.4± 15.6	68.6± 17.3				
Median (min‒max)	70 (30‒90)	70 (30‒90)				
Tonsil enlargement grade, n (%)			0.79	0.27	2.31	0.894^b^
2	2 (50)	2 (50)				
3	24 (60)	16 (40)				
4	7 (63.6)	4 (36.4)				

Statistical tests: Chi-Square

a Student's *t*

b Likelihood-ratio. Sat O_2_, Blood Oxyhemoglobin Saturation.

**Table 3:** SDSC questionnaire subscale findings for the mild OSA and moderate/severe OSA groups.

Variables	OSA		OR	95% CI		p-value
	Mild	Moderate/ Severe		Inferior	Superior	
Disorders in initiating and maintaining sleep			0.98	0.88	1.08	0.619^a^
Mean ± SD	16± 5.9	15.2± 5.1				
Median (min‒max)	15 (7‒31)	14 (9‒28)				
Disorders in breathing during sleep			1.10	0.90	1.33	0.362^a^
Mean ± SD	11.2± 3.2	12± 2.4				
Median (min‒max)	12 (4‒15)	12 (8‒15)				
Disorders of arousal			1.09	0.85	1.41	0.503^a^
Mean ± SD	4.7± 2.4	5.1± 1.7				
Median (min‒max)	4 (3‒13)	5 (3‒9)				
Disorders of sleep-wake transition			1.02	0.91	1.14	0.772^a^
Mean ± SD	16± 4.9	16.4± 4.4				
Median (min‒max)	15 (9‒29)	18 (6‒23)				
Disorders of excessive somnolence			1.01	0.92	1.11	0.812^a^
Mean ± SD	11.5± 6.1	11.9± 5.8				
Median (min‒max)	10 (5‒25)	12.5 (5‒25)				
Nocturnal hyperhidrosis			0.97	0.82	1.16	0.755^a^
Mean ± SD	6.2± 3.1	5.9± 3.3				
Median (min‒max)	7 (2‒10)	6 (2‒10)				
Total			0.99	0.96	1.03	0.649^a^
Mean ± SD	65.9± 15.8	64± 15.7				
Median (min‒max)	61 (47‒106)	60.5 (43‒04)				

Statistical tests: Chi-Squared

a Student's *t*, # Likelihood-ratio.

Comparisons between the mild OSA and moderate/severe OSA groups on the basis of orthodontic and cephalometric analysis are shown in Table 4. Dental characteristic variables were not significantly different between the groups. FD, based on Ricketts cephalometric analysis, showed statistically significant differences (*p* = 0.010) between groups. Table 5 describes the multiple logistic regression model estimated for this variable. A positive association was observed between FD and OSA severity in children (*p* = 0.012). An increase of one degree in the FD angle increased the probability of moderate or severe OSA by 29% (OR = 1.29), as shown in Table 5.

**Table 4:** Orthodontic evaluation and cephalometric analysis of mild and moderate/severe OSA groups.

Variables	OSA		OR	95% CI		p-value
	Mild	Moderate/ Severe		Inferior	Superior	
Lip sealing absence						0.723^a^
No	7 (70)	3 (30)	1.00			
Yes	26 (57.8)	19 (42.2)	1.71	0.39	7.46	
Facial profile						0.509
Plane	15 (55.6)	12 (44.4)	1.00			
Convex	18 (64.3)	10 (35.7)	0.69	0.24	2.05	
Increased overjet						0.437
No	24 (57.1)	18 (42.9)	1.00			
Yes	9 (69.2)	4 (30.8)	0.59	0.16	2.23	
Distocclusion						0.197
No	20 (54.1)	17 (45.9)	1.00			
Yes	13 (72.2)	5 (27.8)	0.45	0.13	1.53	
Transversal arch relationship						0.081^b^
Normal	24 (54.5)	20 (45.5)	1.00			
Unilateral cross bite	5 (71.4)	2 (28.6)	0.48	0.08	2.75	
Bilateral cross bite	4 (100)	0 (0)	d			
Ricketts analysis						
Facial axis (degree)			1.10	0.95	1.28	0.186^c^
Mean ± SD	84.8± 4.1	86.2± 3.7				
Median (min‒max)	85.5 (74.1‒92.1)	86.1 (80.4‒94.1)				
Facial depth (degree)			1.29	1.06	1.57	**0.010**^c^
Mean ± SD	84.1± 4.6	87± 2.8				
Median (min‒max)	84.6 (65.9‒90.7)	86.8 (82.5‒92.5)				
Mandibular plane angle (degree)			0.90	0.79	1.02	0.086^c^
Mean ± SD	32.4± 5.5	30.1± 4				
Median (min‒max)	32 (21.8‒47.2)	30.4 (21.6‒40.4)				
Lower facial height (degree)			0.91	0.80	1.03	0.126^c^
Mean ± SD	51.1± 4.6	49± 4.8				
Median (min‒max)	51.2 (41.9‒64.3)	49.2 (39.4‒60.2)				
Mandibular arch (degree)			0.99	0.94	1.05	0.787^c^
Mean ± SD	24.4± 12.4	23.6± 6.1				
Median (min‒max)	22.1 (11.7‒84.6)	24.4 (11.7‒33.8)				
Facial pattern classification						0.367^b^
Mesofacial	4 (40)	6 (60)	1.00			
Dolichofacial	27 (64.3)	15 (35.7)	0.37	0.09	1.52	
Brachyfacial	2 (66.7)	1 (33.3)	0.33	0.02	5.03	
Jarabak analysis						
Posterior facial height (mm)			1.00	0.91	1.11	0.937^c^
Mean ± SD	55.5± 5.6	55.6± 4.9				
Median (min‒max)	54.4 (46.8‒69.7)	55.9 (47.3‒64.2)				
Anterior facial height (mm)			1.01	0.94	1.08	0.880^c^
Mean ± SD	94.6± 7.8	94.9± 8.1				
Median (min‒max)	93.6 (79.5‒113.7)	94.3 (78.5‒107.5)				
Jarabak index (%)			0.98	0.86	1.13	0.820^c^
Mean ± SD	58.6± 4.4	58.3± 3.7				
Median (min‒max)	59 (50‒68)	58 (51‒67)				
Growth pattern						0.887^b^
Neutral	13 (61.9)	8 (38.1)	1.00			
Hypodivergent	4 (66.7)	2 (33.3)	0.81	0.12	5.50	
Hyperdivergent	16 (57.1)	12 (42.9)	1.22	0.38	3.87	

Statistical tests: Chi-Squared

a Fisher's exact

b Likelihood-ratio

c Student's *t*

d Impossible to estimate. mm, Millimeters.

**Table 5:** Multiple logistic regression model for facial depth.

Variable	OR	95%CI		*p*-value
		Inferior	Superior	
Facial depth	1.29	1.06	1.57	0.012

Statistical test: multiple logistic regression.

## Discussion

Accurate OSA diagnosis in children is best accomplished by careful integration of the results of clinical and anatomical evaluations and PSG values.

The present data showed a lack of association between the OSA severity and the findings of demographic and anthropometric evaluations. Population ethnicity must be taken into account while evaluating these findings, and the genetic diversity in the Brazilian population may be an interference factor in the outcomes. The present anthropometric evaluation results are not in accordance with previous studies that reported obesity as one of the most risk factors not only in children but also in adults.^20^

The authors also found a lack of association between adenotonsillar hypertrophy and OSA severity. This finding was in accordance with the 2019 American Academy of Otorhinolaryngology clinical guidelines,^21^ which reported that children with small tonsils may have severe OSA symptoms and that children without apparent OSA symptoms may have tonsillar hypertrophy and/or nasal airway obstruction. In the authors’ opinion, this probably occurs because OSA severity is related to multiple factors such as craniofacial anatomy and neuromuscular tone and not only upper airway obstruction.

Assessment of OSA symptoms is an important element for diagnosis, and some sleep questionnaires generally show a good correlation with PSG results. However, according to Wise et al.,^8^ most of these questionnaires do not provide strong evidence to support their validity. The authors found no difference between the mild OSA and moderate/severe group in assessments based on sleep symptoms using the SDSC questionnaire. Caregiver reports of snoring witnessed apnea episodes or other nocturnal symptoms may be unreliable if the caregiver does not directly observe the child while sleeping or observes the child in only the early evening.^21^ Therefore, the authors hypothesize that it is difficult to distinguish sleep symptoms in relation to OSA severity because questionnaires and clinical interviews are subjective instruments and are also affected by parental bias.

Absence of lip sealing, hyperdivergent growth pattern, increased MP, increased AF, mandibular retrusion, increased overjet, and increased transverse deficiency are commonly reported as major changes caused by oral breathing.^22^, ^23^, ^24^ Teleradiography and cephalometric analysis is a well-recognized tool for screening dental, skeletal, and soft tissue characteristics and is part of orthodontic documentation. Some authors performed studies seeking to use a non-invasive method for screening craniofacial phenotype, not involving X-Ray imaging in children.^25^^,^^26^ Clinical facial photography is feasible to obtain and shows preliminary evidence of relationships to sleep disorders but other studies are necessary to explore the real value in predicting the risk of OSA.^25^ According to Ikavalko et al.^26^ it would be advantageous if other healthcare professionals, and not only orthodontists, could be trained to play a key role in identifying certain craniofacial risk features for OSA. Photography may provide a complementary assessment but cephalometry is still the best way to evaluate craniofacial characteristics.

There are few published studies in the literature addressing associations between dental and craniofacial deformities and OSA severity.^27^^,^^28^ The authors choose Ricketts's and Jarabak's cephalometric analysis because both are routinely used to establish craniofacial growth pattern, which is constant during life. In a systematic review and meta-analysis, Flores-Mir et al.^27^ found an association between craniofacial characteristics and pediatric OSA, but Katyal et al.^28^ stated that there was no evidence of a direct causal relationship. Di Francesco et al.^29^ found that craniofacial characteristics such as dolichocephaly, mandibular plane, and FD correlate with OSA severity but only in boys. Other studies have reported that dental changes associated with a long face and a vertical growth pattern characterizes the most common OSA phenotype in children.^30^

The present data showed that there was no association between dental characteristics and OSA severity. Differently from what was expected, the findings showed a positive association between FD and OSA severity, but this measurement by itself does not express the child's growth pattern, as it is established by the arithmetic mean of the differences between the obtained angles and the normal values of five cephalometric measurements. The authors hypothesize that the severity of OSA in children may be more influenced by the maxilla position and not by growth pattern or mandibular position as cited in the literature.

## Conclusion

In conclusion, the clinical criteria and craniofacial characteristics evaluated did not influence the disease severity. In the authors’ opinion, it is still difficult to define whether dental and anatomical changes are derived from birth or adaptations from impaired breathing. It is possible that different associations would be observed in younger or older children due to the airway obstruction period.

Nevertheless, as OSA severity is related to multiple factors, it is essential to continue focusing on the diagnosis and evaluating not only mandibular but also maxillary morphology prior to surgery. Choice of treatment must be tailored to the individual child depending on disease severity, predisposing factors, and comorbidities.^31^ Recent literature evidence that OSA management requires a multidisciplinary approach in order to make an early diagnosis and a correct treatment plan.^32^ The interdisciplinary work from health professionals is essential for a good OSA treatment approach in children.

## Study limitations and future studies

Additional prospective multicenter studies using larger samples and including children of different ages and races are required to validate these findings.

It is important to continue studying the effectiveness of other less expensive alternatives to diagnose and predict OSA severity in children.

## Conflicts of interest

The authors declare no conflicts of interest.
